# Simultaneous Dispersive Liquid–Liquid Microextraction and Determination of Different Polycyclic Aromatic Hydrocarbons in Surface Water

**DOI:** 10.3390/molecules27238586

**Published:** 2022-12-05

**Authors:** Zaual Temerdashev, Surendra Prasad, Tatiana Musorina, Tatiana Chervonnaya, Zhanna Arutyunyan

**Affiliations:** 1 Department of Analytical Chemistry, Faculty of Chemistry and High Technologies, Kuban State University, Stavropolskaya St. 149, 350040 Krasnodar, Russia; 2 School of Agriculture, Geography, Environment, Ocean and Natural Sciences (SAGEONS), Discipline of Biological and Chemical Sciences, The University of the South Pacific, Suva, Fiji

**Keywords:** dispersive liquid–liquid microextraction, polycyclic aromatic hydrocarbons, priority pollutants, water samples

## Abstract

Polycyclic aromatic hydrocarbons (PAHs) are a class of persistent organic pollutants of water, and their determination at trace levels in the aquatic ecosystems is essential. In this work, an ultrasound-assisted dispersive liquid–liquid microextraction (DLLME) procedure was suggested utilizing a binary dispersive agent for recovery of different molecular weight polycyclic aromatic hydrocarbons (PAHs) from waters. The detection was carried out by gas chromatography–mass spectrometry (GC-MS) as well as high-performance liquid chromatography with fluorescence and diode-array detection (HPLC-FD/PDA). The method was optimized for the extraction of analytes with respect to the mixture composition, ratios of components, ultrasonication time and centrifugation parameters. The analytical schemes for PAHs extraction from water samples using different ratios of extraction and dispersive solvents are reported. The mixture consisting of chloroform and methanol was applied for the extraction of PAHs containing two or three fused aromatic rings; the mixture of chloroform and acetonitrile is suitable for PAHs containing more than four aromatic rings. The mixture of chloroform:acetone + acetonitrile was applied in the universal scheme and allowed for the simultaneous extraction of 20 PAHs with different structures. The developed sample preparation schemes were combined with GC-MS and HPLC-FD/PDA, which allowed us to determine the analytes at low concentrations (from 0.0002 µg/L) with the recoveries exceeding 80% and relative standard deviations of about 8%. The developed methods for the determination of 20 PAHs were applied to the analysis of water samples from the Karasun Lake (Krasnodar), Azov Sea (Temryuk) and Black Sea (Sochi).

## 1. Introduction

Polycyclic aromatic hydrocarbons (PAHs) are related to eco-toxicants due to their mutagenic and carcinogenic nature [1]. Therefore, the United States Environmental Protection Agency (US-EPA) has recommended the routine monitoring of 16 PAHs in environmental samples. Dibenz[a,h]anthracene, benz[e]pyrene, benz[a]anthracene, benz[b]fluoranthene, benz[k]fluoranthene and inden[1,2,3-c,d]pyrene are the most toxic PAHs. However, less dangerous PAHs can have a synergetic effect when present in a complex mixture. Toxic equivalency factors (TEF) in combination with the maximum permissible concentration (MPC) of individual PAHs in water express the toxicity of complex mixtures in the USA and European Union (EU). TEF for different priority PAHs is expressed as benz[a]pyrene equivalents, and the most toxic substances have the TEF value of 1 [2]. 

Besides PAHs’ differentiation based on toxicity, it is essential to consider their distribution in the water bodies [3,4]. PAHs can be conditionally categorized into three groups based on their water solubility (S) and vapor pressure (P) [5]. The first group corresponds to PAHs containing two to three fused aromatic rings with P of 10^−2^–10^−3^ Pa and water solubility from 2 to 35 mg/L. The second group includes PAHs containing three or four aromatic rings with P of 10^−2^–10^−3^ Pa and solubility between 0.1 and 2 mg/L. The third group consists of PAHs with more than four aromatic rings, vapor pressure less than 10^−5^ Pa and solubility below 0.01 mg/L (Table 1). Thus, it can be assumed that lighter PAHs, such as naphthalene, acenaphthene, fluorene, etc., will be present in the water bodies in dissolved form [6]. On the other hand, the high molecular weight toxic PAHs containing more than four aromatic rings can aggregate on the surface of different particles and precipitate into the sediments [7]. Consequently, it is necessary to consider the transformation and distribution of PAHs in waters [8,9]. With the increase in the molecular weight, the octanol/water partition coefficient (Kow) changes from 3.2 (light PAHs) to 7.1 (heavy PAHs), which shows the tendency of PAHs to accumulate in the lipophilic phase. Naphthalene usually evaporates from the surface of water bodies during 50–200 h depending on natural factors. The evaporation of biphenyl from the surface can take from 7.5 days for surface water to 14 days for groundwater, while the evaporation of acenaphthylene varies with seasonal and climatic factors and can range from 42 to 120 days. In addition, since the degradation of PAHs in water depends on several factors, such as light intensity, time, climatic conditions and microorganisms, the correlation between degradation speed and the number of aromatic rings of PAHs has not been established. It should be considered that this parameter can vary for almost all the components in a wide range from several hours to months and in some cases can even reach years. Thus, fast and precise methods are required for the determination and ecological monitoring of different molecular weight PAHs in water samples within a wide linear range including trace levels and allowing one to account for the degradation of these substances.

As a rule, chromatographic methods, such as gas chromatography–mass spectrometry (GC-MS) and high-performance liquid chromatography (HPLC) with a fluorescence detector (FD), or their combination in complicated cases, are used to quantify PAHs in water [10,11,12]. However, the sensitivity of GC-MS and HPLC-FD can be insufficient for the determination of trace PAH levels in complex matrices without proper sample pretreatment. To solve this problem, it is necessary to apply an effective sample preparation technique providing high recoveries and concentration factors of the analytes [11,13,14].

There exist numerous solid-phase extraction methods for the recovery and concentration of organic pollutants from environmental samples [15,16]. However, liquid–liquid extraction (LLE) methods are more widely used for these purposes due to their availability and simplicity. Combining these methods with ultrasound treatment accelerates mass transfer and consequently increases effectiveness of the processes [17]. A procedure consisting of ultrasound-assisted LLE with *n*-hexane and GC-MS detection has been suggested by us for the extraction and quantification of PAHs from different types of water samples [18]. The procedure provided high recoveries (90%) of PAHs; however, this method is time- and labor-consuming and has high organic solvent consumption.

Dispersive liquid–liquid microextraction (DLLME) seems to be a perspective technique [19,20,21] free from the above-mentioned disadvantages. In this technique, the chlorinated solvents are used as extractants, while acetone, acetonitrile, methanol, etc., are used as dispersive agents. Cloudy solution formation provides a high surface area between the extraction mixture and sample, which results in quick and effective extraction of PAHs from water samples [22,23,24]. The separation of extractant is usually performed by centrifugation [22]. In addition, shaking and ultrasound-assisted extraction techniques were suggested to improve the extraction efficiency [24,25].

In recent years, environmentally friendly solvents have also become attractive for the development of “green” chemistry. In this way, surfactants, low-toxic brominated and other derivates of hydrocarbons [26], deep eutectic solvents (DES) [27,28], ionic liquids (ILs) [29], etc., have been utilized in the developed methods to decrease toxicity of systems. On the other hand, the large-scale use of ionic liquids results in significant material costs and difficulties in understanding their mechanism of action due to the lack of studies. High viscosity of ILs can lead to a decrease in the mass transfer efficiency [30], while the hygroscopicity and absorption of moisture from atmospheric air limit the application of ILs and DES for the extraction of analytes from water samples and make these solvents poorly compatible with chromatographic systems [31]. It should also be noticed that ionic liquids are non-biodegradable [32], which complicates their application for “green” chemistry purposes. The use of deep eutectic solvents in DLLME is complicated by their hydrophilicity. In this case, sample pretreatment is more cumbersome, since it is necessary to apply a multi-step extraction procedure of analytes or properly choose hydrophobic components [28,33]. It can be assumed that a wide application of ionic liquids and deep eutectic solvents in DLLME with chromatographic detection is quite problematic due to insufficient study of their properties. To avoid these shortcomings, it is necessary to conduct a supplementary investigation and develop a conventional DLLME procedure for efficient extraction of PAHs from water samples. The combination of chlorinated solvents and conventional dispersive agents, such as acetone and acetonitrile, provides high extraction efficiency of individual PAHs, e.g., 96% for naphthalene group PAHs, 98% for PAHs with three and four aromatic rings and 95% for PAHs containing more than four aromatic rings [34,35,36,37,38]. However, the behavior of co-existing PAHs with diverse molecular weights in the extraction procedures with conventional solvents was not discussed in these works. The difference in volatility and solubility of particular PAH groups can influence their behavior in the extraction systems. It is essential to consider the problem of the simultaneous microextraction of individual PAHs with significantly different solubilities and vapor pressures from waters. Moreover, no data are available on the investigation of PAHs behavior in the DLLME extraction mixtures and applying a binary solvent mixture as a dispersive agent for these purposes.

In this work, the peculiarities of the dispersive liquid–liquid microextraction and concentration of different molecular weight PAHs with individual conventional solvents (acetone, methanol, acetonitrile) and their binary mixtures as dispersive agents followed by GC-MS and HPLC-FD/PDA detection were investigated.

## 2. Experiment

### 2.1. Reagent and Standards

In total, 17 polycyclic aromatic hydrocarbons (PAHs)—naphthalene (Naph), 2-methylnaphthalene (2-MN), biphenyl (Biph), acenaphthylene (Acy), acenaphthene (Ace), fluorene (Flu), phenanthrene (Phe), anthracene (Anth), fluoranthene (Fluor), pyrene (Pyr), benz[b]fluoranthene (B[b]F), benz[k]fluoranthene (B[k]F), benz[a]anthracene (B[a]A), chrysene (Chry), benz[a]pyrene (B[a]P), dibenz[a,h]anthracene (D[a,h]A) and benz[g,h,i]perylene (B[g,h,i]P)—in acetonitrile with a concentration of 100 or 200 µg/mL of each PAH were purchased from Ecros (St. Petersburg, Russia). Triphenylene (Triph) (analytical standard, 98.8% purity), benz[e]pyrene (B[e]P) and inden[1,2,3,-c,d]pyrene (I [1,2,3,-c,d]P) in cyclohexane were obtained from Sigma-Aldrich, USA (100 µg/mL). Methanol (gradient grade, ≥99.8%) was purchased from Avantor Performance Materials Poland S.A. (Poland). Acetonitrile (gradient grade, ≥99.9%), dichloromethane (ACS reagent, ≥99.5%), tetrachloroethane (ACS reagent, ≥99.0%), carbon tetrachloride (≥99.5%) and acetone (suitable for HPLC, ≥99.8%) were purchased from Sigma-Aldrich, USA, and Merck, Germany. Chloroform (ACS reagent, ≥99.8%) was obtained from Acros Organic, Belgium. Ultrapure water was obtained from a Milli-Q system (Millipore, Bedford, MA, USA).

### 2.2. Samples

Real surface water samples with different salinity and matrices were used to assess the applicability of the developed method for the analysis of real samples. Lake water was collected from the local Lake Karasun (Krasnodar, Russia). Sea water samples were obtained from the Azov Sea (Temruk, Russia) and the Black Sea (Sochi, Russia). The blank water was used for the model samples. All water samples were stored in brown glass bottles and kept at 4 °C prior to analysis.

### 2.3. Instruments

In this work, two chromatographic systems were used for the determination of PAHs: a gas chromatograph with a split/splitless injector coupled to a quadrupole mass spectrometer (Shimadzu GCMS-QP2020, Shimadzu, Japan) and a Shimadzu LC-30 Nexera high-performance liquid chromatograph (Shimadzu, Japan) with an SPD-M30A photodiode array detector (PDA) and an RF-20A/20Axs fluorescence detector (FD) equipped with an autosampler. A Liston C 2201 centrifuge (Russia) was used for phase separation. The compounds were identified using Wiley8 and NIST-17.1 mass spectral libraries as well as the retention times of the individual PAHs standards.

### 2.4. GC-MS Analysis

The injection port temperature was 250 °C, and a split ratio of 1:10 was applied. The oven temperature program was as follows: the initial temperature was 60 °C, then it increased from 60 °C (held for 1 min) to 170 °C at the rate of 15 °C/min, from 170 °C (held for 3 min) to 280 °C at the rate of 10 °C/min, from 280 °C(held for 8 min) to 290 °C at the rate of 10 °C/min and held constant at 290 °C for 25 min. The ion source temperature was maintained at 250 °C. Ultra-pure helium (99.995%) was used as a carrier gas at a constant linear velocity of 30 cm/s. Selected ion monitoring (SIM) mode was used to achieve higher sensitivity and minimize the influence of matrix. Separation of 20 PAHs including difficult to separate isomers, such as phenanthrene/anthracene, chrysene/triphenylene/benz[a]anthracene, benz[b]fluoranthene/benz[k]fluoranthene and benz[a]pyrene/benz[e]pyrene, was carried out on a 5 ms Zebron capillary column (60 m × 0.25 mm, 0.25 µm). The integration of target analyte peaks on the chromotogram was performed by the GCMSsolution software Version 4.45. 

### 2.5. HPLC-FD/PDA Analysis

To separate the PAHs, a Kinetex 3.5 µm PAH column (150 × 4.5 mm) was used. Deionized water and acetonitrile were used as eluents in the gradient elution mode as follows: the initial eluent composition was 50% acetonitrile for 3 min; 3–10 min: a linear ramp to 100% acetonitrile with a plateau at 100% acetonitrile for 8 min; 18–18.5 min: a decrease to 50% acetonitrile; 18.5–20 min: a plateau at 50% acetonitrile for 1.5 min; the flow rate was 1 mL/min. Since acenaphthylene does not fluoresce, PDA was used for its detection at the wavelength of 254 nm. Detection was performed by programming the excitation and emission wavelengths to obtain better sensitivity and minimize interferences. The excitation/emission wavelength pairs (nm) were as follows: 0.01–7.50 min: 280/325 nm; 7.50–8.90 min: 265/380 nm; 8.90–9.70 min: 290/420 nm; 9.70–20 min: 300/500 nm. The column was thermostated at 35 °C. The total HPLC run time was 20 min. The software LabSolutions Version 5.73 was used for the integration of the target analyte peaks on the chromatogram. 

### 2.6. Extraction Procedure

Based on the literature data [36,37] and results obtained in our laboratory, the extractant for DLLME was selected. Chloroform, dichloromethane, dichloroethane and carbon tetrachloride were tested as extraction solvents. Acetonitrile was used as a dispersive solvent in the initial experiments, because it provided stable and reproducible extraction systems. To evaluate the effect of extraction solvent type, a series of sample solutions were tested using 1 mL of acetonitrile and 50, 100, 150, 200 and 500 µL of chlorinated solvents for water sample volumes of 10 mL. Experiments were carried out by spiking model water samples with PAH standard solutions at different concentration levels, i.e., low (0.0002 µg/L), medium (0.1 µg/L) and high (1 µg/L). The experiments were carried out in three replicates. Based on the obtained results, three procedures for the extraction of various PAHs from water have been developed. 

Extraction of light PAHs. In a glass centrifuge tube, 10 mL of water was added, and the extraction mixture consisting of 150 µL of chloroform and 400 µL of methanol was quickly injected by using a 2 mL syringe. Then, the tube was shaken for 1 min and ultrasonicated for 2 min at 35 kHz. To separate the sample and extractant (chloroform) phases, the tube was centrifuged for 2 min at 2600 rpm.

Extraction of heavy PAHs. In a glass centrifuge tube, 10 mL of water was added, and the extraction mixture (150 µL of chloroform and 1.5 mL of acetonitrile) was quicky introduced into the sample by using a 2 mL syringe. Then, the tube was shaken for 1 min and centrifuged for 5 min at 3600 rpm for phase separation.

Extraction of 20 PAHs. In a glass centrifuge tube, 10 mL of water was quickly spiked with the extraction mixture, i.e., 150 µL of chloroform and 1.0 mL of acetonitrile + acetone (1:1) binary dispersive agent, by using a 2 mL syringe. Then, the extraction system was shaken for 1 min and ultrasonicated for 6 min at 35 kHz. For the phase separation, the tube was centrifuged for 2 min at 2600 rpm. 

## 3. Results and Discussion

### 3.1. Estimation of Effectiveness of DLLME Extraction Solvents for the Recovery of PAHs from Water

**Figure 1:**
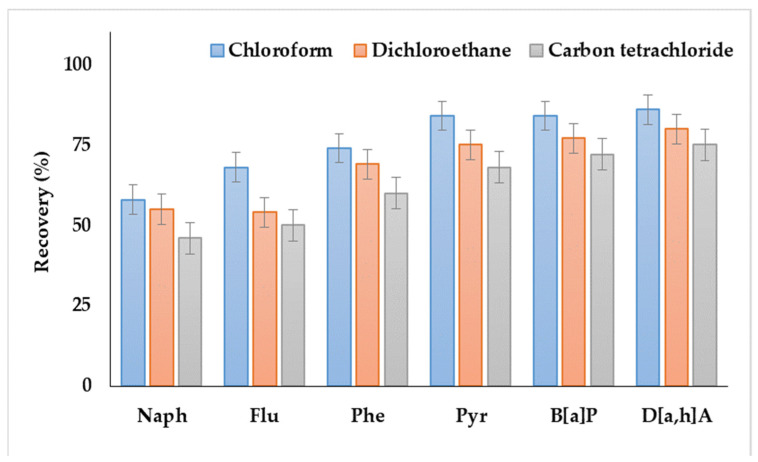
Comparison of polycyclic aromatic hydrocarbon (PAH) recoveries from water samples by using various extraction solvents.

As shown in Figure 1, the systems containing dichloroethane or carbon tetrachloride as extractants provided the lowest recoveries. Moreover, the extractant phase was not separated in the case of dichloromethane. Satisfactory analyte recoveries were achieved by using chloroform, which agreed with the results of our previous study, i.e., the application of DLLME for the extraction of PAHs from soils and bottom sediments [38].

**Figure 2:**
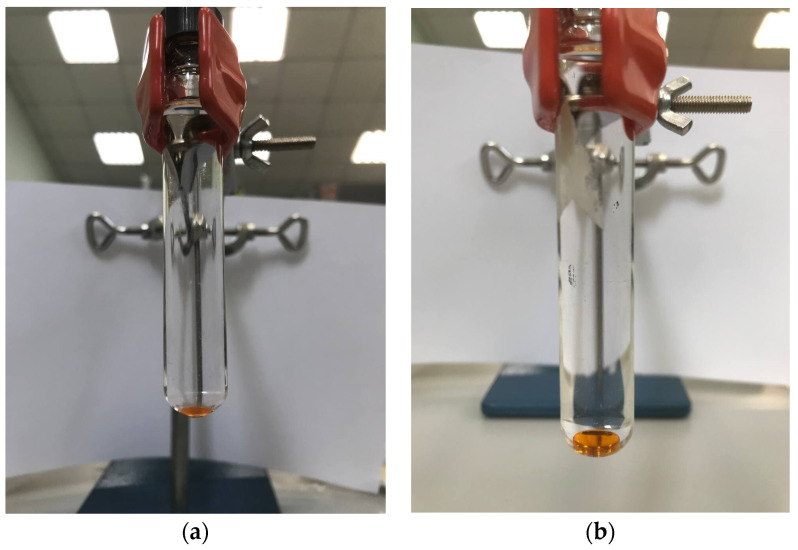
Stability of the extraction system for the recovery of PAHs using chloroform in volume: (**a**) 100 µL; (**b**) 150 µL.

At this stage, the use of chloroform volumes less than 150 µL was established to result in the insufficient extractant phase separation (Figure 2a). With further increase in chloroform volume, a decrease in the sensitivity was observed. A stable extraction system and sufficient recoveries were obtained by using 150 µL of chloroform (Figure 2b) and, consequently, this extractant volume was chosen as optimum. 

When the ratio of a tested sample to solvent volume is selected, several requirements should be considered: the volume of water has to be sufficient for the effective concentration of analytes and the quantitative separation of chloroform drop from the extraction system. The last condition limits the maximum sample volume. According to these limitations, the sample volume of 10 mL was chosen in the following extraction steps.

Based on the obtained results, chloroform volume of 150 µL and 10 mL of water sample were used in further experiments.

### 3.2. Selection of Dispersive Solvent and It Is Optimal Volume

The selection of appropriate combinations and ratios of dispersive and extraction solvents is essential because it affects the formation of a stable and effective extraction system. Acetonitrile, acetone, methanol and their binary mixtures like acetone + acetonitrile, methanol + acetone and methanol + acetonitrile were chosen as dispersive agents. The extraction mixtures with a constant volume of the extractant (150 µL of chloroform) were used in the following investigation, and volumes of dispersive solvents were between 0.2 and 1.7 mL. The criteria for selecting the volume of the dispersive solvent were the formation of a cloudy solution and minimum dissolution of the extractant in the dispersive solvents in water phase (the necessary and sufficient conditions). The extraction systems that were satisfactory reproducible in terms of droplet volume in replicate experiments were chosen for further tests (Table S1). Based on experimental results, the optimal volumes of individual dispersive solvents were selected, and for acetonitrile it was 1.5 mL, for methanol it was 0.4 mL and for acetone it was 1.0 mL. Among the binary solvent mixtures, stable systems were obtained only when acetonitrile volume was equal or exceeded the other solvent volume (methanol or acetone). In the case of acetone and methanol mixture, utilization of excessive acetone volumes allowed us to obtain stable extraction systems. Thus, it was 0.5 + 0.5 mL for mixtures containing acetone and acetonitrile or acetone and methanol, and 0.75 + 0.75 mL of the mixture with methanol and acetonitrile. These dispersive solvent volumes were used in the following stage. The extraction capability of the developed solvent mixtures by PAHs was assessed by analysis of water samples spiked with PAHs at two concentration levels (5 and 50 ng/mL). 

**Figure 3:**
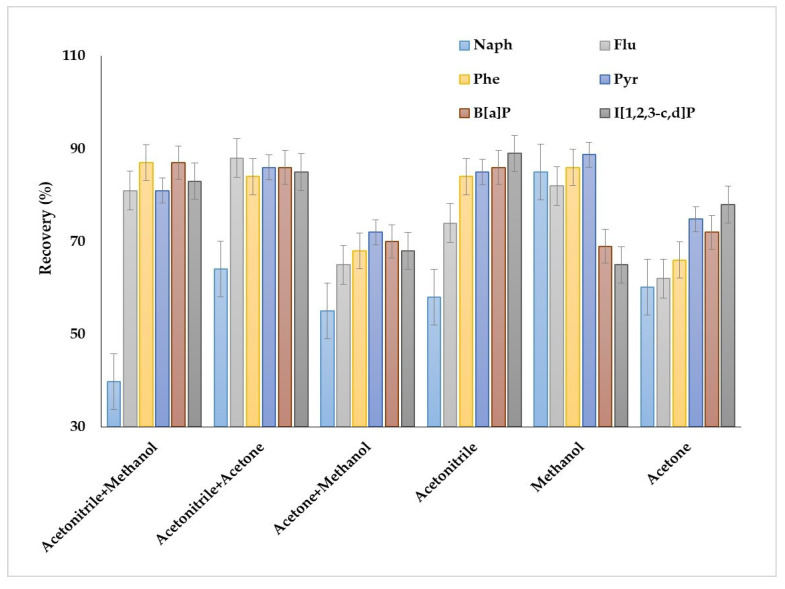
Effect of the dispersive agent compositions on the extraction recovery of PAHs.

The obtained results demonstrated several peculiarities of PAHs extraction when individual solvents were used as dispersive solvents (Figure 3). Acetonitrile was preferable for the extraction of heavy and several light PAHs (fluorene, phenanthrene, anthracene, fluoranthene, pyrene); for these compounds, the recoveries were up to 89%. However, the recoveries of naphthalene group PAHs (acenaphthene, acenaphthylene, biphenyl and 2-methylnaphthalene) were less than 60%. Application of methanol allowed us to increase the recoveries of naphthalene group PAHs to 78–85% and provided high extraction efficiency for fluorene, phenanthrene, anthracene, fluoranthene and pyrene (80–89%). Acetone showed the worst results as a dispersive solvent among the solvents. In this case, the maximum recoveries were 75–76% for benz[b]fluoranthene, benz[k]fluoranthene and inden[1,2,3–c,d]pyrene, while the recoveries were less than 65% for other compounds; therefore, acetone was not used in the further studies.

Thus, the results demonstrated the possibility of highly efficient extraction of particular groups of PAHs by individual dispersive solvents, i.e., acetonitrile for the extraction of PAHs containing three or more aromatic rings and methanol for light PAHs. However, the individual dispersive agents did not provide simultaneous efficient extraction of a large list of PAHs. The application of binary mixtures as the dispersive agents was studied to solve this problem. The binary mixtures of acetonitrile with methanol or acetone provided high extraction recovery of PAHs containing more than three aromatic rings. The recoveries were comparable in both cases and ranged between 82 and 89%. However, the mixtures were ineffective for the extraction of naphthalene group PAHs; for example, the recoveries of naphthalene by methanol + acetonitrile and acetone + acetonitrile were 43 and 64%, respectively. The application acetone + methanol mixture resulted in obtaining the lowest extraction recoveries of all compounds.

It can be stated that the individual dispersive solvents provide potentially high extraction recoveries for light PAHs or the analytes containing three or more aromatic rings, and it is possible to develop a universal effective scheme for the simultaneous recovery of different molecular weight PAHs by applying the binary mixture consisting of acetone and acetonitrile.

To increase PAHs recoveries, the influence of ultrasound treatment and centrifugation parameters on the developed extraction mixtures was investigated.

### 3.3. Ultrasonication Effect on Extraction of PAHs from Water

**Figure 4:**
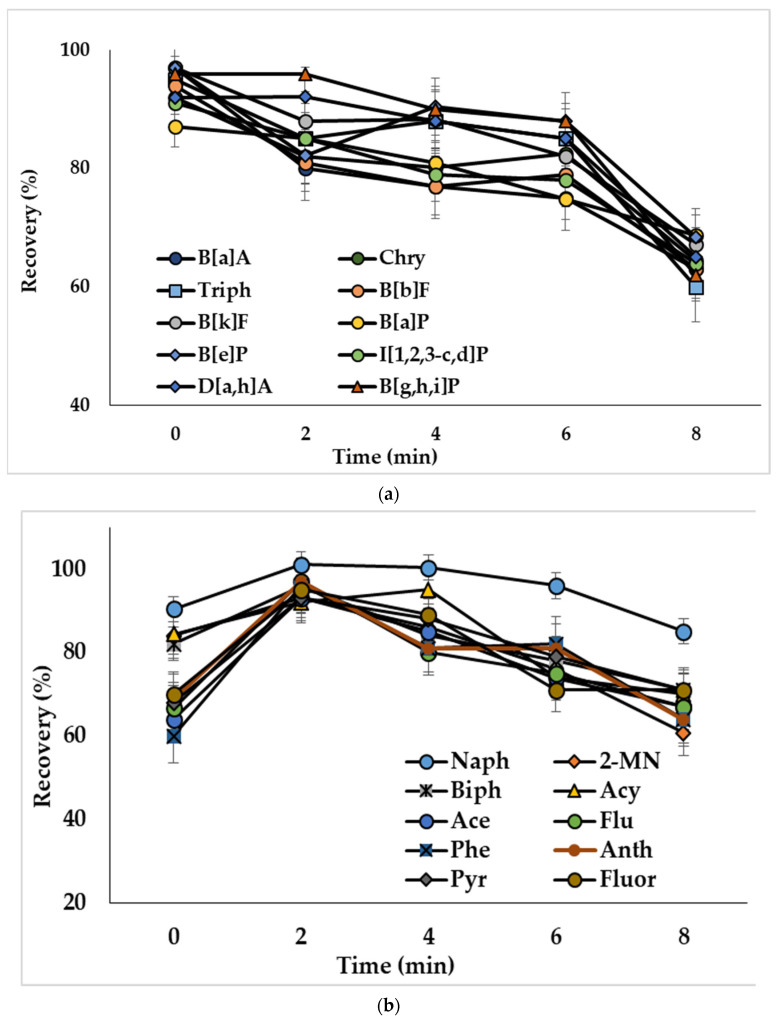

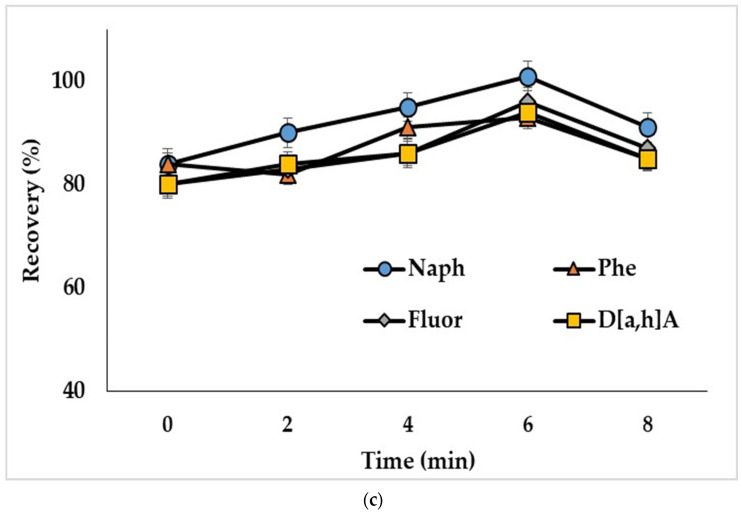
Effect of ultrasonication time on the recoveries of PAHs with extraction mixture: (**a**) chloroform and acetonitrile (mixture I); (**b**) chloroform and methanol (mixture II); (**c**) chloroform and acetone + acetonitrile (mixture III). Concentration of the analytes: 2.5 ng/mL.

The ultrasound treatment in DLLME provides decreased size of solvent drops and, consequently, increases the extraction efficiency [39]. To study this influence, water samples spiked with PAHs at 50 ng/L (concentration of analytes in the extract was 2.5 ng/mL) were ultrasonicated from 2 to 8 min. Ultrasonication of the extraction mixture containing acetonitrile as a dispersive solvent (mixture I) resulted in decreased recoveries of compounds (Figure 4a). The observed effect could be related to the reverse mass transfer of the analytes from chloroform to acetonitrile-water phase. Ultrasonication showed a positive effect on the extraction of analytes, when mixtures with methanol (mixture II) and the binary dispersive agent (mixture III) were used, and the optimal treatment times were 2 min (Figure 4b) and 6 min (Figure 4c), respectively. The effect of ultrasonication on the extraction of representative PAHs by the mixture containing binary dispersive agent is shown in Figure 4c; similar results were obtained for other investigated compounds.

### 3.4. Effect of Centrifugation on Extraction of PAHs from Water

The selection of centrifugation parameters is also important for the formation of the extractant drop. To study the effects of centrifugation speed and time on the recoveries of PAHs, the following conditions were tested: 2600 rpm (2 min), 3000 rpm (2 min), 3000 rpm (5 min), 3000 rpm (10 min) and 3600 rpm (5 min). Water samples spiked with PAHs at 50 ng/L (concentration of analytes in the extract was 2.5 ng/mL) were used in the experiments (3 replicates). 

The results of experiments showed that best conditions for mixture I were 3000 and 3600 rpm for 5–10 min. In this case, the recoveries exceeded 99% for each PAH. However, the mixtures II and III had different behavior at long centrifugation times and high speed, and the extraction recoveries of PAHs were decreased. The optimized centrifugation conditions for mixtures II and III were 2600 rpm and 2 min. The optimization of ultrasonication and centrifugation conditions allowed us to increase the extraction efficiency of analytes by using the three suggested mixtures. 

Based on obtained results, three extraction schemes were suggested for the sample preparation of water by using DLLME with physical effects: procedure A for the extraction of PAHs containing more than three fused aromatic rings; procedure B for the extraction of naphthalene group PAHs and PAHs containing no more than four aromatic rings; a universal procedure C for the extraction different PAHs. For procedure A, in the first step, mixture I was added to the water sample. Next, the samples were shaken and centrifuged for 5 min at 3600 rpm to separate the extractant. For procedures B and C, after the addition of mixture II or III to the water sample, shaking and ultrasonication of the systems were conducted for 2 or 6 min, respectively. Centrifugation was carried out for 2 min at 2600 rpm to separate the extractant. Under the optimized conditions, procedure A provided the recoveries of heavy PAHs of 95–102% depending on the analyte (from fluorene to benz[g,h,i]perylene); the recoveries of light PAHs (from naphthalene to pyrene) were 95–101% by using procedure B; the recoveries between 91 and 99% for PAHs were obtained by procedure C (Table 2).

The universal DLLME procedure with the binary mixture (acetone + acetonitrile) as the dispersive agent was validated, because it provided high recoveries in the simultaneous extraction of a large list of PAHs.

### 3.5. Determination of Different PAHs by Dispersive Liquid–Liquid Microextraction and GC-MS and HPLC-FD/PDA Detection

**Figure 5:**
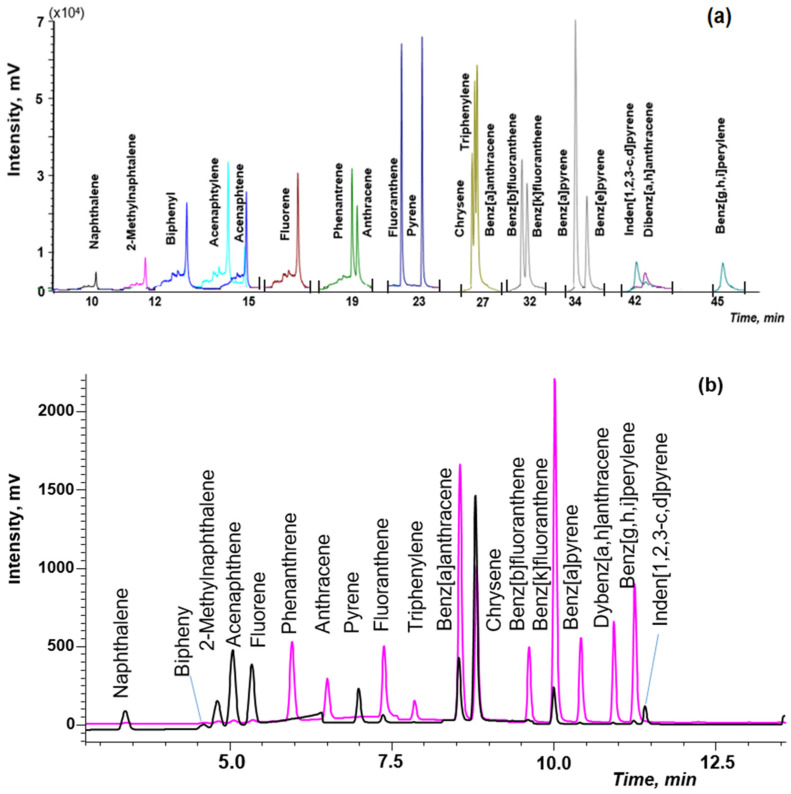
The chromatogram of the standard PAHs solution (50 ng/mL) by: (**a**) gas chromatography and mass spectrometry (GC-MS); (**b**) high-performance liquid chromatography and fluorescence detector (HPLC-FD).

The combination of DLLME with GC-MS is the simplest option, because chloroform can be directly injected into GC-MS after centrifugation. Previously optimized conditions of the GC-MS detection of PAHs in soils [38] were used in this work. The separation of isomeric pairs, such as phenanthrene/anthracene, chrysene/triphenylene/benz[a]anthracene, benz[b]fluoranthene/benz[k]fluoranthene and benz[a]pyrene/benz[e]pyrene, was achieved by using the temperature program: from 60 °C to 290 °C at the three stages on a specialized capillary column (Figure 5a). Application of the selected ion monitoring (SIM) mode allowed us to increase sensitivity and reliability of PAHs determination in complex matrices of natural waters. 

However, to determine lower concentrations of PAHs in waters, the application of HPLC-FD/PDA is advisable. The separation PAHs was achieved by using the gradient elution and time program of FD (Figure 5b).

The chromatographic conditions described in EPA 8310 method and work [40] were used as initial to optimize HPLC-FD determination of PAHs. However, when these conditions were applied for the separation of 20 PAHs at the flow rate of 1.5 mL, peaks overlapped; at the flow rate of 0.5 mL, the HPLC performance was insufficient. The flow rate of 1 mL/min under gradient elution conditions and timing program of the fluorescence detector allowed us to solve the problem. Since acenaphthylene does not fluoresce, the PDA detector was used at the wavelength of 254 nm for the determination of this analyte. 

The combination of DLLME with HPLC-FD/PDA requires evaporation of chloroform and re-extraction of the residue in a solvent compatible both with the chromatographic system and PAHs; acetonitrile was selected for this purpose. It is well known that naphthalene group PAHs with high vapor pressure can evaporate during this process [5]. In our work, naphthalene, 2-methylnaphthalene, biphenyl, acenaphthene, acenaphthylene and fluorene losses did not exceed 8–11%, while for other PAHs, the losses were lower than 4%, when chloroform was evaporated under the stream of nitrogen (water samples were spiked with PAHs at low, medium and high concentration levels, 3 replicates).

### 3.6. Analytical Performance and Real Water Analysis

The developed method was validated in terms of linearity, limit of detection (LOD), limit of quantitation (LOQ) and precision. As can be seen in Table S2, good linearity of calibration curves for GC-MS and HPLC-FD/PDA detection was established, and the determination coefficients (R^2^) were >0.99. The LODs for all PAHs were calculated as the signal/noise (S/N) ratios of 3, and they were in the range of 0.10–0.20 ng/L for HPLC-FD/PDA and between 10 and 20 ng/L for GC-MS. To check the intra-day assay precision of the developed method, 16 replicate samples with concentrations of individual PAHs of 30 ng/L (HPLC-FD/PDA) and 100 ng/L (GC-MS) were extracted with the DLLME method with binary solvents (acetone + acetonitrile) as dispersive agents and analyzed within one day. The inter-day assay precision was assessed at 30 ng/L (HPLC-FD/PDA) and 100 ng/L (GC-MS) spiking concentration during 10 consecutive days (Table S2). The relative standard deviations (RSDs) for the target PAHs were in the ranges of 3.1–6.5% and 3.7–7.8% (intra-day, HPLC-FD/PDA and GC-MS, respectively) and 4.3–7.0% and 5.4–8.2% (inter-day, HPLC-FD/PDA and GC-MS, respectively). It may be concluded that the combination of the universal sample preparation scheme with the chromatographic detection method can be selected depending on the required sensitivity of PAHs determination. The combination of DLLME with GC-MS is advisable for the determination of PAHs at concentrations above 10 ng/L in waters with complex matrices due to the increased reliability of PAHs identification. For trace concentrations of PAHs (0.10–0.20 ng/L), DLLME should be combined with HPLC-FD/PDA.

The validated method for the determination of PAHs was applied to different types of water samples. Table 3 shows the results of real sample analyses. To evaluate the matrix effect of the validated method, the spike-recovery test was performed by spiking 20 PAHs at three concentration levels (0.2, 10 and 750 ng/L) into the tap water sample and at two concentration levels of 10 and 750 ng/L into lake (salinity of 0.5 ‰) and seawater samples (the Azov Sea salinity of 6–18 ‰ and the Black Sea salinity of 22 ‰). As can be seen in Table 3, the recoveries for the PAHs in real water samples were in the range from 88 to 103%, and the RSDs were in the range of 3.1–7.8%, demonstrating good precision. Satisfactory repeatability of measurements proved that the sample matrices and salinity of water negligibly affected the determination of PAHs; consequently, the developed method can be applied to the analysis of different types of waters. Furthermore, the developed method allows for determining naphthalene, 2-methylnaphthalene, biphenyl and acenaphthene in waters at trace levels.

## 4. Estimation of the Extraction Effectiveness of PAHs from Waters with Different DLLME Types

The proposed method for the extraction of 20 PAHs and DLLME concentration with the binary dispersive agent and HPLC-FD/PDA detection was compared with the previously reported methods (Table 4). For comparison, the following procedures were selected: DLLME with tetrachloroethylene as an extractant and acetone as a dispersive agent [19], low-density solvent-based DLLME (LDS-DLLME) with acetonitrile as a dispersive agent and hexane as an extractant [41], vortex-assisted DLLME (VSA-DLLME) technique with dichloromethane as an extractant and vortex mixing for the dispersion [24] and solidification of deep eutectic solvent DLLME (DLLME–SFDES) using deep eutectic solvent as an extractant and ultrasonication for the dispersion [42]. It should be noted that all the procedures have comparable recoveries; however, the proposed procedure is more universal regarding different PAHs. Meanwhile, the developed method has lower LODs for most analytes in comparison with other methods [19,24,41,42]. The suggested approach for the optimization of analyte extraction recoveries covers the differences between physicochemical properties of particular PAH groups more comprehensively. It allows us to improve the analysis scheme and choose the optimal parameters for the simultaneous recovery and concentration of components followed by their trace quantification. 

The developed method is fast and precise, which is proved by accuracy values lower than 8% that are reproducible with the relative standard deviation values of 4.3–7.0%, thus allowing researchers to use it in analytical laboratories for environmental monitoring.

## 5. Conclusions and Future Perspectives

In the present paper, different water sample preparation schemes were developed for the determination of PAHs based on DLLME concentration and HPLC-FD/PDA or GC-MS detection. Extraction solvent mixtures containing individual dispersive solvents (methanol or acetonitrile) provided high recoveries (>95%) for light PAHs or the analytes containing three or more aromatic rings. However, this sample preparation scheme is not universal when PAHs with different molecular weights are simultaneously determined. DLLME with acetone and acetonitrile as a binary dispersing solvent in combination with ultrasonication allowed us to achieve higher recoveries of a large list of PAHs from water samples (>99%), as they are compatible with chromatographic detection methods. With the relative standard deviation of 3.1–7.8%, GC-MS provided detection and quantification limits of 3.0–6.0 and 10–20 ng/L, respectively. In the case of HPLC-FD/DAD determination of PAHs, the detection and quantification limits were 0.03–0.07 and 0.10–0.20 ng/L, respectively. Analysis of water samples with different mineralization has shown that salinity has negligible effect on PAH recoveries.

The developed analysis schemes allowed us to determine PAHs with different structures in water samples within a wide concentration range. The analysis schemes are suitable for routine environmental monitoring, do not require highly qualified personnel and minimize the volumes of used chloroform, which agrees with “green” analytical chemistry.On the other hand, despite the mentioned advantages of the DLLME procedure for the concentration of the analytes, its shortcoming is the use of a toxic chlorinated solvent, even in small volumes. The aim of subsequent studies is to find biodegradable solvents with comparable analyte concentration factors.

## Figures and Tables

**Table 1:** Some physicochemical properties of polycyclic aromatic hydrocarbons.

PAHs			Mr	S, mg/L	P, Pa	K_ow_	Half-Life in the Environment, h
Light	2 to 3 rings	Naphthalene	128	31–34	37–42	3.2–3.8	16–6193
		2-Methylnaphthalene	142	20.0–27.3	6.3–10.7	3.9–4.1	54–9840
		Biphenyl	154	7.0–7.8	1.3–6.9	3.2–4.3	36–336
		Acenaphthylene	152	3.4–16.1	0.89–1.1	3.7–4.1	1020–1440
		Acenaphthene	154	3.9–3.8	0.21–3.1	3.9–4.5	3–4896
	3 to 4 rings	Fluorene	166	1.6–1.8	0.08–0.79	3.7–4.3	768–2880
		Phenanthrene	178	1.0–1.2	0.02–0.11	4.5–4.6	3–9600
		Anthracene	178	0.04–0.08	5.7 × 10^−4^–0.1	4.2–5.3	1–22,080
		Pyrene	202	0.1–0.2	1.7 × 10^−4^–0.2	4.8–5.5	1–91,200
		Fluoranthene	202	0.21	1.3 × 10^−4^–0.1	4.5–5.2	21–21,120
Heavy	more than 4 rings	Benz[a]anthracene	228	0.0090–0.0094	3.9 × 10^−7^–2.5 × 10^−4^	5.0–5.9	0.5–32,640
		Chrysene	228	0.0015–0.0170	8.4 × 10^−7^–2.3 × 10^−4^	5.5–5.9	0.5–48,000
		Triphenylene	228	0.040	3.9 × 10^−7^–1.2 × 10^−2^	4.8–6.3	–
		Benz[b]fluoranthene	252	0.0011–0.0015	5.0 × 10^−8^–6.7 × 10^−5^	5.8	9–29,280
		Benz[k]fluoranthene	252	0.0008–0.0011	1.3 × 10^−8^–6.7 × 10^−5^	5.9–7.2	4–102,720
		Benz[a]pyrene	252	0.0016	7.5 × 10^−7^–1.1 × 10^−4^	6.0–8.0	0.4–25,440
		Benz[e]pyrene	252	0.0001–0.0073	7.4 × 10^−7^–1.8 × 10^−5^	5.7–7.4	–
		Inden[1,2,3,-c,d]pyrene	278	0.0002–0.0004	1.3 × 10^−8^–1.3 × 10^−7^	6.7–8.2	–
		Dibenz[a,h]anthracene	276	0.0006–0.0025	3.7 × 10^−10^–2.5 × 10^−7^	5.8–7.1	6–45,120
		Benz[g,h,i]perylene	276	0.0001–0.0008	1.3 × 10^−8^–6.7 × 10^−7^	6.2–7.1	14,160–31,200

Mr—Molecular weight. S—Water solubility at 25 °C. P—Vapor pressure. K_ow_—Octanol/water partition coefficient.

**Table 2:** The recoveries of PAHs by using different sample preparation procedures.

PAHs	Recovery (%)		
	Procedure A	Procedure B	Procedure C
Naph	74 ± 5	101 ± 4	91 ± 5
2-MN	75 ± 4	99 ± 4	92 ± 3
Biph	75 ± 5	99 ± 4	92 ± 4
Acy	74 ± 5	98 ± 3	92 ± 5
Ace	76 ± 4	100 ± 3	93 ± 4
Flu	95 ± 3	100 ± 8	92 ± 5
Phe	97 ± 3	99 ± 4	93 ± 3
Anth	97 ± 4	95 ± 4	93 ± 4
Pyr	98 ± 4	101 ± 7	95 ± 3
Fluor	99 ± 4	98 ± 4	92 ± 4
B[a]A	100 ± 6	82 ± 6	98 ± 4
Chry	98 ± 3	82 ± 6	98 ± 3
Triph	98 ± 3	80 ± 5	95 ± 4
B[b]F	100 ± 4	79 ± 4	96 ± 4
B[k]F	98 ± 3	78 ± 4	97 ± 4
B[a]P	98 ± 4	76 ± 3	97 ± 4
B[e]P	102 ± 5	73 ± 3	98 ± 4
I [1,2,3-c,d]P	101 ± 4	71 ± 3	98 ± 3
D[a,h]A	98 ± 3	72 ± 3	98 ± 3
B[g,h,i]P	99 ± 3	72 ± 3	99 ± 3

**Table 3:** Results of HPLC-FD/PDA determination of PAHs in real water samples (n = 2, *p* = 0.95).

Analyte	Sample												
	Tap Water				Lake Water			Sea Water ^1^			Sea Water ^2^		
	Mean (ng/L)	ER + RSDs (%)			Mean (ng/L)	ER + RSDs (%)		Mean (ng/L)	ER + RSDs (%)		Mean (ng/L)	ER + RSDs (%)	
		Spicked Amount (ng/L):				Spicked Amount (ng/L):			Spicked Amount (ng/L):			Spicked Amount (ng/L):	
		0.2	10	750		10	750		10	750		10	750
Naph	<0.20	91 ± 7.7	92 ± 7.5	95 ± 7.5	<0.20	88 ± 7.5	91 ± 7.1	<0.20	91 ± 7.4	95 ± 7.7	<0.20	93 ± 7.1	95 ± 7.2
2-MN	<0.15	92 ± 6.2	93 ± 6.3	93 ± 6.0	<0.15	90 ± 7.2	92 ± 7.5	<0.15	92 ± 7.7	94 ± 7.8	<0.15	92 ± 7.5	97 ± 7.7
Biph	<0.15	93 ± 6.2	92 ± 6.9	94 ± 6.8	<0.15	85 ± 6.8	86 ± 6.1	<0.15	95 ± 6.5	96 ± 6.8	<0.15	92 ± 7.6	95 ± 7.8
Acy	<0.15	94 ± 6.5	95 ± 6.4	95 ± 6.3	<0.15	87 ± 5.8	89 ± 6.5	<0.15	96 ± 6.5	99 ± 6.6	12	93 ± 6.1	96 ± 6.3
Ace	<0.15	93 ± 4.1	94 ± 3.8	95 ± 3.6	<0.15	87 ± 3.7	91 ± 3.5	<0.15	89 ± 4.0	92 ± 4.2	<0.15	94 ± 4.3	97 ± 4.5
Flu	<0.15	93 ± 5.0	95 ± 4.7	98 ± 4.5	<0.15	92 ± 4.7	91 ± 4.8	1.6	99 ± 4.5	95 ± 4.3	<0.15	92 ± 4.1	95 ± 4.3
Phe	<0.15	92 ± 3.9	99 ± 3.7	98 ± 3.5	3.1	87 ± 3.3	90 ± 2.9	1.4	96 ± 3.5	94 ± 3.7	1.3	99 ± 3.5	98 ± 3.6
Anth	<0.15	93 ± 5.1	95 ± 4.9	93 ± 4.7	0.46	98 ± 3.8	95 ± 3.7	<0.15	92 ± 3.6	95 ± 3.8	<0.15	95 ± 3.8	97 ± 4.1
Pyr	<0.15	95 ± 3.8	98 ± 3.5	95 ± 3.4	2.2	101 ± 3.1	98 ± 3.2	<0.15	93 ± 3.5	96 ± 3.4	14	94 ± 3.3	98 ± 3.2
Fluor	<0.15	96 ± 4.1	98 ± 4.0	96 ± 4.0	5.9	93 ± 3.8	95 ± 3.6	0.72	96 ± 3.7	98 ± 3.5	0.19	97 ± 3.7	99 ± 3.5
B[a]A	<0.10	95 ± 3.8	97 ± 3.7	95 ± 3.5	<0.10	98 ± 3.5	96 ± 3.6	<0.10	94 ± 3.2	95 ± 3.4	<0.10	97 ± 3.6	101 ± 3.8
Chry	<0.10	96 ± 4.5	98 ± 4.0	96 ± 4.1	<0.10	99 ± 4.1	97 ± 3.9	0.11	91 ± 4.0	96 ± 4.2	0.27	102 ± 4.0	98 ± 3.9
Triph	<0.10	95 ± 4.6	95 ± 4.4	95 ± 4.1	<0.10	97 ± 4.0	95 ± 4.1	0.84	89 ± 4.5	95 ± 4.3	5.7	96 ± 4.4	98 ± 4.6
B[b]F	<0.10	94 ± 4.7	95 ± 4.4	94 ± 4.2	0.78	92 ± 4.1	95 ± 4.2	<0.10	91 ± 4.3	94 ± 4.5	0.19	94 ± 4.1	99 ± 4.4
B[k]F	<0.10	95 ± 4.1	96 ± 4.0	95 ± 4.0	0.35	103 ± 3.9	99 ± 3.8	<0.10	103 ± 3.5	98 ± 3.7	0.1	98 ± 3.6	101 ± 3.8
B[a]P	<0.10	95 ± 4.0	96 ± 3.8	95 ± 3.7	<0.10	96 ± 3.8	97 ± 4.0	<0.10	93 ± 3.5	95 ± 3.7	<0.10	95 ± 3.3	99 ± 3.5
B[e]P	<0.10	95 ± 4.5	97 ± 4.4	95 ± 4.2	1.8	92 ± 4.1	95 ± 4.3	<0.10	92 ± 4.5	96 ± 4.0	0.18	95 ± 4.4	98 ± 4.6
I [1,2,3-c,d]P	<0.10	96 ± 3.5	98 ± 3.3	96 ± 3.2	<0.10	98 ± 3.1	101 ± 3.0	<0.10	96 ± 3.5	99 ± 3.2	<0.10	97 ± 3.7	99 ± 3.9
D[a,h]A	<0.10	97 ± 3.7	95 ± 3.5	97 ± 3.2	3	103 ± 3.2	99 ± 3.3	<0.10	96 ± 3.6	97 ± 3.7	0.27	96 ± 3.5	99 ± 3.7
B[g,h,i]P	<0.10	96 ± 3.4	95 ± 3.5	96 ± 3.3	0.21	105 ± 3.2	101 ± 3.1	<0.10	96 ± 3.5	98 ± 3.7	0.12	92 ± 3.3	97 ± 3.5

^1^—The Azov Sea; ^2^—The Black Sea; ER—extraction recovery; RSDs—relative standard deviations; mean—the average resulted of analytes concentration in the real water samples.

**Table 4:** The LODs (ng/L) and recoveries (%) of PAHs using various DLLME techniques.

PAHs	n-DLLME ^a^ GC-FID * [19]		VSA-DLLME ^b^ GC-MS [24]		LDS-DLLME ^c^ GC-MS [41]		SFDES–DLLME ^d^ HPLC-FD [42]		UA-DLLME ^f^ HPLC-FD/PDA **	
	LOD	ER	LOD	ER	LOD	ER	LOD	ER	LOD	ER
Naph	10	99	2	82	58	85	6.6	103	0.07	91
2-MN	n/d	n/d	n/d	n/d	n/d	n/d	n/d	n/d	0.05	92
Biph	n/d	n/d	n/d	n/d	n/d	n/d	n/d	n/d	0.05	92
Acy	10	97	2	82	43	93	n/d	n/d	0.05	92
Ace	7	82	2	83	34	92	n/d	n/d	0.05	93
Flu	8	92	2	85	23	98	1.2	97	0.05	92
Phe	9	99	2	82	28	93	3.4	83	0.05	93
Anth	9	95	2	83	26	99	0.7	103	0.05	93
Pyr	10	91	3	84	37	96	0.9	107	0.05	95
Fluor	10	111	2	83	38	102	4.3	93	0.05	92
B[a]A	10	103	3	76	46	83	n/d	n/d	0.03	98
Chry	10	94	3	81	49	83	n/d	n/d	0.03	98
Triph	n/d	n/d	n/d	n/d	n/d	n/d	n/d	n/d	0.03	95
B[b]F	n/d	n/d	2	77	n/d	n/d	n/d	n/d	0.03	96
B[k]F	n/d	n/d	3	74	n/d	n/d	n/d	n/d	0.03	97
B[a]P	15	102	3	84	n/d	n/d	n/d	n/d	0.03	97
B[e]P	20	102	n/d	n/d	n/d	n/d	n/d	n/d	0.03	98
I [1,2,3-c,d]P	n/d	n/d	5	78	n/d	n/d	n/d	n/d	0.03	98
D[a,h]A	n/d	n/d	5	78	n/d	n/d	n/d	n/d	0.03	98
B[g,h,i]P	30	101	5	74	n/d	n/d	n/d	n/d	0.03	99

LOD—limit of detection; ER—extraction recovery of PAHs at concentration of each analyte: ^a^ 5 µg/L; ^b^ 0.2 µg/L; ^c^ 0.5 µg/L; ^d^ 0.3 µg/L; ^f^ 0.2 µg/L. n/d—no data; * FID—flame ionization detector; ** developed method HPLC-FD/PDA determination of PAHs.

## Data Availability

The data presented in this study are available on request from the corresponding author.

## Supplementary Materials

The following supporting information can be downloaded https://www.mdpi.com/article/10.3390/molecules27238586/s1, Table S1: The results obtained for different extraction systems containing chloroform for DLLME; Table S2: Analytical performance of the DLLME with binary solvents as dispersive agents for the determination of PAHs in waters.
