# Supplementary material for: Simultaneous Dispersive Liquid–Liquid Microextraction and Determination of Different Polycyclic Aromatic Hydrocarbons in Surface Water
Source: Molecules. 2022 Dec 5;27(23):8586. doi: 10.3390/molecules27238586 (PMC9736002; doi:10.3390/molecules27238586)
Supplement: Supplementary file 1 [file molecules-27-08586-s001.zip › molecules-2038697-supplementary.pdf]

Supplementary materials

Table S1. The results obtained for different extraction systems containing chloroform for DLLME.

No	Dispersive agent	Volume, mL	Observation
1	Acetonitrile	0.5	lack of drop
2		1.0	unstable drop
3		1.2	
4		1.5	stable drop
5		1.7	unstable drop
6	Methanol	0.2	stable drop
7		0.4	
8		0.5	unstable drop
9		1.0	lack of drop
10		1.5	
11	Acetone	0.5	lack of drop
12		0.6	unstable drop
13		0.8	
14		1.0	stable drop
15		1.2	lack of drop
16	Acetone+acetonitrile	1.0 + 0.5	lack of drop
17		0.50 + 0.50	stable drop
18		0.33 + 0.66	
19		0.25 + 0.75	
20	Methanol+acetonitrile	1.0+0.5	lack of drop
21		0.75+0.75	stable drop
22		0.5+1.0	
23		0.4+1.1	
24	Methanol+acetone	1.0+0.5	lack of drop
25		0.5+0.5	stable drop
26		0.2+0.8	
27		0.2+1.0	
28		0.5+1.0	

Table S2. Analytical performance of the DLLME with binary solvents as dispersive agent for the determination of PAHs in waters.

PAHs	LOD, ng/L		LOQ, ng/L		Linearity range , ng/L		Intra-day precision (n=16)		Inter day precision (n=10)	
	HPLC-FD/ PDA	GC-MS	HPLC-FD/ PDA	GC-MS	HPLC-FD/ PDA	GC-MS	HPLC-FD/ PDA	GC-MS	HPLC-FD/ PDA	GC-MS
Naph	0.07	6.0	0.20	20	0.20 – 1000	20 – 7500	6.5	7.1	7.0	8.2
2-MN	0.05	4.5	0. 15	15	0.15 – 1000	15 – 7500	6.5	7.8	5.5	7.6
Biph	0.05	4.5	0.15	15	0.15 – 1000	15 – 7500	5.1	6.5	5.8	6.7
Acy	0.05	4.5	0.15	15	0.15 – 1000	15 – 7500	4.9	5.2	5.2	5.8
Ace	0.05	4.5	0.15	15	0.15 – 1000	15 – 7500	4.2	4.5	5.3	6.1
Flu	0.05	3.0	0.15	10	0.15 – 1000	10 – 7500	5.1	5.5	5.2	6.2
Phe	0.05	3.0	0.15	10	0.15 – 1000	10 – 7500	3.1	3.7	4.8	6.3
Anth	0.05	3.0	0.15	10	0.15 – 1000	10 – 7500	4.2	4.0	4.7	5.8
Pyr	0.05	3.0	0.15	10	0.15 – 1000	10 – 7500	3.3	3.9	4.6	5.7
Fluor	0.05	3.0	0.15	10	0.15 – 1000	10 – 7500	3.8	3.7	4.5	5.9
B[a]A	0.03	3.0	0.10	10	0.10 – 750	10 – 7500	4.4	4.0	4.3	5.8
Chry	0.03	3.0	0.10	10	0.10– 750	10 – 7500	4.1	4.5	4.5	5.4
Triph	0.03	3.0	0.10	10	0.10– 750	10 – 7500	3.9	4.2	4.4	5.5
B[b]F	0.03	3.0	0.10	10	0.10– 750	10 – 7500	4.5	5.1	5.2	6.0
B[k]F	0.03	3.0	0.10	10	0.10– 750	10 – 7500	4.3	5.2	4.5	5.9
B[a]P	0.03	3.0	0.10	10	0.10– 750	10 – 7500	4.2	5.3	5.3	5.8
B[e]P	0.03	3.0	0.10	10	0.10– 750	10 – 7500	4.3	5.0	5.1	6.1
I[1,2,3-c,d]P	0.03	3.0	0.10	10	0.10– 750	10 – 7500	3.4	3.8	4.7	5.8
D[a,h]A	0.03	3.0	0.10	10	0.10– 750	10 – 7500	3.3	3.9	4.8	5.9
B[g,h,i]P	0.03	3.0	0.10	10	0.10– 750	10 – 7500	3.2	3.7	4.5	5.5
